# Physical and Mental Health of Informal Carers from Culturally and Linguistically Diverse (CALD) and Non-CALD Groups in Australia

**DOI:** 10.3390/healthcare12202072

**Published:** 2024-10-17

**Authors:** Rafat Hussain, Danish Ahmad, Rahul Malhotra, Mary Ann Geronimo

**Affiliations:** 1 School of Medicine and Psychology, Australian National University, Canberra 0200, Australia; danish.ahmad@anu.edu.au; 2 Duke-NUS Medical School, Singapore 169857, Singapore; rahul.malhotra@dukenus.edu.sg; 3 Federation of Ethnic Communities’ Councils of Australia (FECCA), Canberra 2601, Australia; maryann@fecca.org.au

**Keywords:** informal carers, CALD, physical health, mental health, Australia

## Abstract

Introduction: Empirical evidence shows that many family carers, especially immigrants, experience considerable health disadvantages and poorer quality of life. Australia has a rapidly increasing multicultural population, officially referred to as Culturally and Linguistically Diverse (CALD) people. This paper explores similarities and differences in the carer profile and physical and mental health of CALD and non-CALD family carers. Methods: A cross-sectional anonymous survey was conducted of self-reported family carers aged 18 years and older. Identical paper and online survey modes were provided to enable choice. Key variables included demographic and carer profile, diagnosed chronic physical health conditions, and validated scales such as CESD-12 and MOS-SF12, including derivative composite Physical and Mental Component Summary (PCS and MCS, respectively) scores. The sample comprised 649 participants (CALD = 347, non-CALD = 302). The analyses included univariate, bivariate, and multivariable linear regression analyses for three outcome variables: PCS, MCS, and CESD-12. Results: CALD carers were comparatively younger and married, and 54% had university-level education (29% in the gfvnon-CALD group). Women were primary carers in both groups (67.4% versus 72.2%). The weekly care hours were higher for non-CALD carers. Both groups had below population-referenced scores for mean PCS and MCS values. For CESD-12, non-CALD respondents had higher scores (17.5 vs. 11.2, *p* < 0.022). Regression analyses showed significant differences for demographic, carer, and physical health variables across the three outcome variables. Discussion and Conclusion: Women have a higher domestic workload, which, when combined with high care hours, adversely impacts physical and mental health. The need for improved and culturally aligned care support systems is required.

## 1. Introduction

An ageing and growing population in Australia is accompanied by a rise in unpaid caregiving. Typically, caregiving for other family individuals who experience long-term health conditions is usually provided by different family members in a caring or unpaid capacity. Such caregivers are called family or informal carers, as formal carers receive financial support for care provision. In Australia, the available estimates show that there are over 2.65 million informal carers who care for family members and close friends [1,2]. This figure equates to one in nine Australians. It is estimated that the cost equivalent of informal care is AUD 77.9 billion annually, or 1% of the total GDP of Australia [3].

Empirical evidence from international studies shows that many informal carers, compared to non-carers, experience considerable health, social, and economic disadvantages, as well as poorer quality of life [4,5,6,7]. A recent systematic review of population-based studies of comparison between carers and non-carers found higher levels of ill-health, such as generalised perceived pain, spinal issues, and, in particular, back pain, as well as mental health issues, including anxiety, depression, and insomnia [8]. Many Australian studies have concentrated on caregiving for health conditions, which are more debilitating and stressful for informal carers, such as palliative care for cancers and dementia [9,10,11]. However, other studies of informal carers, irrespective of the care recipient’s health condition, also report poor health of carers [12,13,14]. While Australia’s proportion of informal carers is increasing, the country also witnesses shifts in the underlying population profile.

Australia, like other countries, has a rapidly changing population profile. In the latest census in 2021, the total population was close to 26 million, with 16% aged 65 years and older [15]. It has one of the highest life expectancies in the world [16]. A large component of the recent population growth in Australia is amongst immigrants due to natural increase and new immigrants. Some historical context regarding immigration is useful before outlining the rationale for this present study. Australia’s rich multicultural society comprises both immigration from other higher-income countries whose native language is not English and skilled-based immigration from low and middle-income countries. There is a rapidly growing CALD Australian population, with an increase of one million immigrants between the two Australian national censuses [15,17]. About 28 percent of people living in Australia were born outside, with about 23% of Australians speaking a language other than English at home [15].

Given the changing Australian demographics to reflect a more nuanced approach to increasing cultural and linguistic diversity, the Australian government made a policy change, and the previous terminology of people from a Non-English-Speaking Background (NESB) was replaced with the term ‘Culturally and Linguistically Diverse communities’ (CALD), reflecting Australia’s multicultural society [18]. The new CALD terminology is based on the language spoken at home, ancestry, and cultural differences. Meanwhile, Australia’s traditional owners/custodians, the Aboriginal and Torres Strait Islander communities, are not considered CALD but a distinct population group with its unique history, heritage, and cultural norms [18]. Therefore, the non-CALD group includes those whose ancestry belongs to early settlers from the late 18–19th centuries, and several subsequent waves of immigrants who were ‘native English speakers’ from the United Kingdom, New Zealand, Canada, USA, and Australia [18].

Although Australia’s immigration policy is geared towards skills-based migration, it also includes a family union pathway allowing parents to join their children. Apart from small humanitarian pathways (for people affected by conflict and war, etc.), the ethnic profile of Australian immigrants has shifted in line with international events from countries in Europe to Southeast Asia and now increasingly from specific Asian countries such as India, China, Nepal, and others. Australia now has a substantial CALD population, and this change is reflected in its listing as one of six priority population groups for future research by state and federal governments [19]. Some people belonging to CALD communities encounter greater challenges when navigating government systems such as health, social welfare, disability care, aged care, etc., due to either not being aware of system-level issues or having language difficulties. This, in turn, may push them towards a greater risk of poorer health outcomes compared to other Australians or non-CALD populations [15,18]. Similar health disparities are also noted for CALD populations in other high-income countries where the acronym CALD may be replaced by another term, such as Black and Asian minority (BAME), minority ethnic groups, and specific cultural groups.

As mentioned, the proportion of Australians classified as informal carers is 2.65 million or 11% of the total population. The latest estimation of the proportion of informal carers in the CALD population is higher, with estimates of 25–30%, which is equivalent to about 500,000 carers. According to Carers Australia, the peak national body of Australian carers, this figure is likely to be an underestimate, as many CALD carers do not identify themselves as ‘carers’, as providing care is integrally linked to familial roles and expectations; hence, many informal carers remain unidentified [20]. Moreover, the pathway for recognition could be missed due to limited access to carer services within CALD communities [21,22].

There are limited recent studies that focus on the health and well-being of informal carers from CALD communities in Australia [23], except for studies on dementia [5,22], and none comparing CALD and non-CALD groups with the same population-based research study. This current paper fills an important population health need by first describing key health and well-being differences between informal caregivers from CALD and non-CALD groups in Australia.

## 2. Materials and Methods

### 2.1. Study Design

This study uses primary data from a cross-sectional study conducted nationally in Australia between September and December 2022. The survey adopted an anonymous questionnaire developed using validated scales and key variables to capture carers’ health status, demographic, and carer characteristics. The questionnaire was reviewed by four external experts in multicultural health in Australia representing academia and community-based organisations and policy. The survey form was pilot tested with a small group of CALD representatives for feedback on ease of understanding the wording of various questions, the relevance of topics, and survey completion time. In the survey pack, each respondent received a survey information summary sheet in their ethnic languages. Based on feedback from pilot testing, some modifications were made to the final questionnaire, such as revising the question on age from ‘age at last birthday’ to 5-year age category, removing questions on postcodes, adding more explanatory notes for various sections such as primary versus secondary carer, and inclusion of open-ended response categories. The potential data loss due to these changes is minimal as the age variable could not be used as continuous variable. Lack of information on postcodes meant that we could not analyse participant location as urban or rural. Note that the majority of the Australian population lives in urban regions. Four grocery vouchers were offered, with winners selected through a lucky dip draw of all completed survey serial numbers. The main study was only undertaken after being approved by the Human Research Ethics Committee of the Australian National University (approval number 2022/074, 29 June 2022)

### 2.2. Study Measures

The survey included questions on physical and mental health, social networks and supports, perception of positive aspects of caregiving, the burden of caregiving, and respite services. In this current paper, the information is limited to participants’ demographic information, basic caregiving characteristics, and physical and mental health, as it is not possible to cover the entire study’s findings within one journal article. Additional journal papers are being developed, which explains the lack of any cross-referencing of findings from the findings of other scales.

To assess carers’ health status, we used validated scales such as the Medical Outcome Study Short Form (MOS-SF-12), the Centre for Epidemiologic Studies Depression 12-item scale (CESD-12), and a list of questions comprising common chronic physical health conditions (see details below). The SF-12 scale is a widely used validated tool derived from the original SF-36 form [24]. It has been used in different populations and yields strong correlations across the eight key domains of the SF-36 scale, with reliable psychometric properties for general health questions, and the two derive physical and mental component summary scores (PCS and MCS) as SF-36 [24,25]. SF-12 has high internal consistency with Cronbach’s alpha, typically above 0.70, with minimal floor and ceiling effects [26,27]. The range for PCS and MCS scores is between 0 and 100, with higher scores indicative of better physical and mental health, with population norms set at 50 [24]. The CESD-12 scale, one of the shorter derivatives of the original CESD-20 scale, is used to assess depressive symptomatology. CESD-12 has high internal consistency and good test–retest reliability, similar to the CESD-20 [28,29]. A score of 16 and above on the CESD-12 scale indicates depressive symptomatology [30,31].

For physical health, the respondents were asked to indicate whether they had a diagnosed medical condition from a list of 14 chronic physical conditions. The list of 14 conditions was drawn from recent government health data on common chronic diseases prevalent in Australia. However, the terminology was kept simple for easier comprehension without changing the disease category. For example, hypertension was listed as high blood pressure, and coronary artery disease as episodic chest pain or heart attack. Additionally, two open-ended response categories were provided for other health conditions not included in the list. We also asked some questions about basic health-seeking behaviour, including consulting a doctor (in person or online) and formally taking leave from work due to ill health.

### 2.3. Survey Recruitment and Data Collection

For the main survey, the sampling strategy comprised digital and in-person approaches with online and print copy distribution. A plain language, detailed ‘Information Sheet’ preceded the survey form by outlining the objectives, types of questions in the survey, a clear statement that completion of the survey meant implied consent (agreement) for providing information, information on how the grocery voucher information could not be linked through the paper or online completed form to the participant, contact details of support agencies for stress associated with their carer role, and Human Research Ethics Committee (HREC) contact details for any complaints regarding the study. A separate project-specific e-mail and mobile number were also listed for general survey queries with the respondents encouraged not to provide personal identifying details unless they belonged to a carer agency wanting to get more information to promote the study. The consent information was clearly listed both in print and online survey forms with prospective participants told that responding to survey questions meant agreeing to provide information. The print survey was replicated online using Qualtrics as a mobile-friendly version and the prospective participants were given the choice to either complete the online survey form (using either the provided weblink or QR code) and the delinked raffle voucher form or complete the printed survey and raffle form and send back the separate reply-paid envelopes included in the survey pack.

Since access to CALD-prospective participants required a multi-prong approach, we initially approached community-based carer organisations such as multicultural councils in various cities to find ‘project facilitators’ to promote the study to ethnic communities. Other agencies such as the Federation of Ethnic Community Councils of Australia (FECCA) [32], Parkinson’s Australia, and Dementia Australia included the study blurb and weblink in their newsletters. This study was also promoted through social media and other platforms, providing a brief overview with a web link and QR code to the online version of the survey to all informal carers—both from CALD and non-CALD communities. Each printed survey pack contained a paper copy of the (a) project blurb, (b) information sheet (as described above), (c) survey form, (d) reply-paid return survey form, (e) a separate raffle form requiring only the first name and street address (which could be a pseudonym and a relative or friend’s postal address), and (f) a separate reply-paid envelope for the completed raffle form. As mentioned earlier, prospective respondents could complete the anonymous survey online or as a paper copy, whichever option they found more convenient, as the investigators were cognisant that paper-based replies required leaving the sealed envelopes at a carer agency office, giving it to one of their volunteers, or getting someone to take the sealed envelopes to a post office to drop them in the mailbox.

### 2.4. Data Analysis

A total of 688 respondents completed the survey, of which most opted to complete the digital version. The completed postal responses comprised only 11%, which indicates a preference for online completion due to convenience of time and not having to find a post office for return mail. Cross-verification was undertaken for data integrity to identify missing and incorrect responses. A few cases (*n* = 35) were removed due to several sections containing incomplete information. Other deletions included a small number where information on the care recipient (who, how many, the relationship) was inconsistent. The final analysis sample comprised 649 respondents, of whom 347 respondents identified themselves as belonging to CALD and 302 from non-CALD communities.

Statistical analyses included univariate and bivariate analysis using standard statistical tests depending on data type (measures of central tendency, chi-square test, and ANOVA) using IBM SPSS statistics (version 27) [33]. Stepwise multivariable linear regression models were developed separately for three outcome variables: SF-12 PCS, SF-12 MCS, and CESD-12 for CALD and non-CALD analysis samples. Before undertaking regression modelling, correlational analysis assessed multicollinearities for all independent variables. Additionally, we also checked for interaction effects. With regards to multicollinearity, a statistically significant correlation was found between education level and adequacy of financial resources, hours of care provision per week, and the number of other carers in the family. Therefore, we chose only one of these variables (e.g., education level and hours of care provision per week) for regression models. Only two interaction terms were statistically significant: carer status by number of care hours per week and carer status by years of care provision.

Stepwise linear regression was undertaken separately for CALD and non-CALD analysis samples for each outcome variable. Each model included four demographic variables (age, sex, marital status, and educational level), four care-provision variables (carer status, number of care recipients, number of reported care hours per week, and duration (in years) of caregiving), interaction terms, and number of chronic physical health conditions. PCS was the dependent (outcome) variable, and MCS and CESD scores were included. Similarly, where MCS was the outcome of interest, PCS and CESD scores were included. Likewise, PCS and MCS scores were included for CESD as the outcome variable. For all regression analyses, VIF was estimated to ensure no additional collinearity. The model summary statistics are R^2^ and Adjusted R^2^, F-test value, and *p*-values were assessed along with standardised β coefficients, t-score, and *p*-values.

## 3. Results

Of the 649 respondents in the analysis sample, 53.4% (*n* = 347) identified as belonging to a CALD group, and the remaining 302 (46.6%) as non-CALD. Information on key demographic variables such as age, sex, marital status, educational level, adequacy of financial resources, country of birth, and, for those not born in Australia, time (in years) since migration are provided in Table 1. The CALD respondents were younger than non-CALD respondents (*p* < 0.05). In both groups, caregiving was heavily gendered, i.e., females were the main family carers (67.4% in the CALD group versus 72.2% in the non-CALD group). Other demographic variables, such as marital status and education (formal years of schooling), showed statistically significant differences (see Table 1). Nearly 60% of the CALD respondents and 12.6% of non-CALD respondents were born overseas (*p* < 0.001). This sub-group of non-CALD respondents were older respondents who may have immigrated from the UK as part of family migration to Australia.

Table 2 includes information on key caregiving characteristics. Family size varied between the two groups, but the mean family size was similar (mean: 3.8 ± SD; 1.4 the in CALD group and 3.8 ± SD, 0.6 in the non-CALD group). In both groups, most respondents were primary carers (CALD = 79.5%, non-CALD = 80.1%), with the rest being secondary carers (Table 2). The number of people for whom care was provided (care recipients) was small (mean: 1.2, SD ± 0.8 for the CALD group; mean: 0.9, SD ± 0.8 for the non-CALD group) (see Table 2). A small proportion (5.1%) of carers reported being involved on a 24/7 basis per week, i.e., providing extensive care for almost every aspect of care provision, including getting up several times at night. Setting aside this small group, the mean number of care hours per week was statistically higher (*p* < 0.001) in the non-CALD group compared to the CALD group. Similarly, there was a statistically significant difference (*p* < 0.001) in the duration of caregiving between CALD and non-CALD carers. A quarter of CALD carers indicated that they need interpreter services for care recipients compared to only 5% of the non-CALD respondents (*p* < 0.001). There is no clear explanation as to why this small group of non-CALD carers indicated a need for interpreter services, except for anecdotal evidence that some people find navigating social care services and, at times, specialist healthcare services daunting and prefer using an interpreter familiar with the appropriate terminology.

In Table 3, the health profile of informal carers is outlined. This includes the number of medically (clinician) diagnosed chronic physical health conditions. Additionally, three variables derived from SF-12, namely general health status and physical component summary (PCS) and mental component summary (MCS) scores, were included. Information on CESD-12 is presented as the total score based on values as a continuous variable and a binary variable using the prescribed cut-off scores. With regards to chronic health conditions, 37% of CALD and 27% of non-CALD carers had no health conditions, whereas 15.6% of CALD and 18.5% had one condition. Although not statistically significant, there were some inter-group differences regarding multimorbidity (the presence of two or more chronic conditions), with 47% of CALD and 54% of non-CALD carers reporting multimorbidity (see Table 3). Responses to the general health question of SF-12 showed statistically significant variation across the two groups. In the CALD group, 10.7% of respondents rated their health as excellent, and the proportions of ‘very good’ and ‘good’ were 23.3% and 40.6%. The corresponding values for the non-CALD group were 6% (excellent), 23.2% (very good), and 35.8% (good). Further analyses to assess the association between self-reported general health and the presence of chronic conditions. The difference in general health was statistically different by the number of chronic physical health conditions in both groups (F = 7.1, df = 4, *p* < 0.001). 

**Figure 1:**
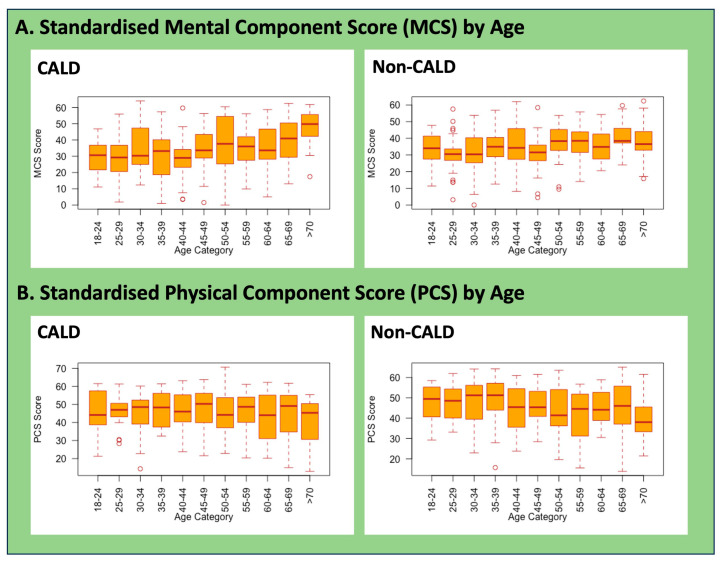
Standardised MCS and PCS values by age between CALD and non-CALD groups. Note: differences in MCS and PCS scores were statistically significant by age and ethnicity.

**Figure 2:**
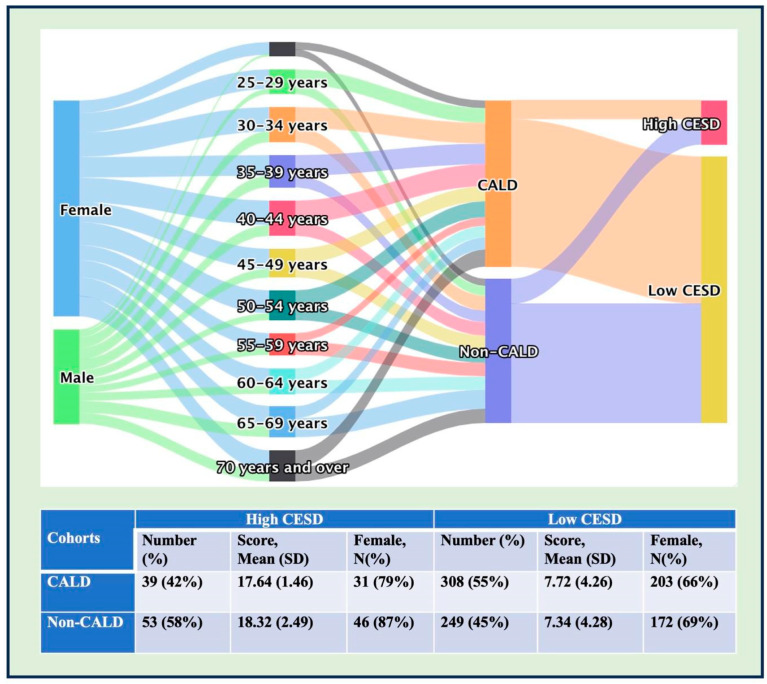
Age, sex, and CESD score differences between carers from CALD and non-CALD groups. Sankey diagram and descriptive statistics of the high and low CESD patterns in CALD and non-CALD cohorts. The non-CALD cohort exhibits a higher percentage (53/302 people; 17.5%) of ‘High CESD’ compared to the CALD cohort (39/347 people; 11.2%).

PCS and MCS scores in both groups were below the population norms (Table 3). The mean PCS scores were 45.5 ± SD 10.6 for the CALD group compared to 44.8 ± SD 11.0 for the non-CALD group (*p* = 0.431). The corresponding MCS values for both groups were 35.1 ± SD, 11.3 compared to 35.6 ± SD, 14.0 (*p* = 0.6–7). The standardised aggregate PCS and MCS scores varied by age for both CALD and non-CALD groups, with some age groups showing statistically significant large-value ranges (see Figure 1). For CESD-12 scores, analysing the scores as a continuous variable showed no statistically significant difference (*p* = 0.607) between the two groups (mean = 8.8, SD ± 5.1 in the CALD group; mean = 9.3 (SD ± 5.8) in the non-CALD group). The scores were also analysed as a dichotomous variable using a cut-off score of 16 or higher, indicating depressive symptomatology. We found a statistically significant difference (*p* = 0.022) with proportionately more non-CALD respondents (53/302, 17.5%) having a ‘high CESD’ score compared to the CALD group (39/347, 11.2%) (see Table 3 and Figure 2). Moreover, from a gender perspective, more females from non-CALD (15%) communities scored higher on the CESD scores compared to females from CALD communities (9%), indicating a higher mental health burden in the former group (see Figure 2). Consultations with one or more doctors for health issues showed some intergroup differences; these were not statistically significant. On the other hand, taking leave from work to manage health issues was found to be statistically significant (*p* = 0.022), with 38.0% of CALD and 20% of non-CALD respondents taking leave.

### 3.1. The Results of Multivariable Linear Regression for Three Outcome Variables

The regression analyses were undertaken separately for the CALD and non-CALD groups. The regression models included all variables listed in Table 1 and Table 2 as explained in the text in the methods and results sections. The interaction terms used for regression have been outlined in the methods section. At the bivariate level, only two interaction terms were statistically significant for CALD and non-CALD samples: “carer status and number of care hours per week” and “carer status and years of care provision”. Both interaction terms were included in regression analyses, but neither was found to be statistically significant in the final regression models. We chose a stepwise regression technique to limit the need for separate tables for each outcome variable, which otherwise would be needed to present the non-significant results for covariates. Note that regression modelling techniques are essentially the same across different methods. The only difference is in the way the final model output is shown—i.e., all variables, irrespective of statistical significance versus only statistically significant variables. The stepwise approach follows the same steps as other linear regression methods, but the final model output only shows statistically significant variables. The regression analysis results for PCS, MCS, and CESD-12 are outlined in Table 4. This table presents key model summary statistics, statistically significant predictor variables, standardised coefficients (stdβ), and t-statistic and *p*-values. The stepwise regression approach allows a generous *p* = 0.20 significance level for inclusion in various iterations of regression analyses with a cut-off of *p* < 0.05 for elimination of a variable from the final model. Hence, only those variables that were found to be statistically significant at *p* < 0.05 are included for presentation in Table 4.

### 3.2. Physical Component Summary Score (PCS)

In the regression model for the CALD group, age, marital status, and hours of care per week were statistically significant. However, none of these variables were found to be statistically significant for the non-CALD group. Also, neither of the interaction terms remained statistically significant in either group after adjusting for other variables. The largest influence on PCS scores appeared to be health conditions in the CALD group (*std*β = −0.39, *p* < 0.001), whereas in the non-CALD groups, it was the CESD score (*std*β = −0.46, *p* < 0.001). The number of chronic physical health conditions was inversely associated with a PCS score for both groups, which indicates some causal association between pre-existing chronic health conditions and PCS scores. Similarly, in both groups of carers, the final regression model showed a statistically significant inverse association for MCS (*std*β = −0.22, *p* < 0.001) for the CALD group and also the non-CALD group (*std*β = −0.29, *p* < 0.001). A similar pattern was observed for CESD scores (Table 4).

### 3.3. Mental Component Summary Score (MCS)

The stepwise regression model had the same demographic, care-provision, and health variables as well as interaction terms as the PCS regression model, except for including PCS as a predictor variable. In this analysis, for both CALD and non-CALD groups, age was found to have a small but statistically significant association with MCS scores (CALD group: *std*β = 0.11, *p* = 0.027; non-CALD: *std*β = 0.15, *p* < 0.001). However, no other demographic and care-provision variables nor a number of chronic physical health conditions were statistically significant. PCS score had a small but inverse association with MCS in each group (CALD: *std*β = −0.18, *p* < 0.001; non-CALD: *std*β = −0.14, *p* < 0.001 (see Table 4)). The strongest influence was the total CESD scores, which showed an inverse association with MCS score (CALD: *std*β = −0.67, *p* < 0.001; non-CALD: *std*β = −0.75, *p* < 0.001).

### 3.4. Centre for Epidemiologic Studies Depression Scale (CESD 12)

The stepwise regression analysis for the total CESD-12 scores showed differences among predictor variables for each regression model. Being a female had a small but statistically significant association for the non-CALD group (*std*β = 0.92, *p* = 0.023) but not for the CALD group. Care-provision variables were only significant for the CALD group and included being a primary carer (*std*β = 0.91, *p* = 0.041) and number of care recipients (*std*β = 0.13, *p* = 0.004). In each group, all three health status predictors influenced the CESD-12 scores. The number of chronic physical health conditions impacted CESD-12 scores (CALD: *std*β = 0.14, *p* = 0.004; non-CALD group: *std*β = 0.89, *p* = 0.022). An inverse association between PCS values and CESD was found in both groups: CALD (*std*β = −0.18, *p* < 0.001) and non-CALD (*std*β = −0.23, *p* < 0.001). The largest influence on CESD-12 scores was from the MCS score in each group (CALD: *std*β = −0.62, *p* < 0.001; non-CALD: *std*β = −0.70, *p* < 0.001 (see Table 4)).

## 4. Discussion

Contextualizing demographic findings, although caregiving happens across all ages, our study found that 40% of the carers in the CALD group and over a third (34.4%) in the non-CALD group were aged between 25 and 44 years. As indicated by other studies [34,35,36], this is a life-stage period where most carers have multiple roles, from establishing a career to having family responsibilities. This life stage also coincides with establishing some economic security. An earlier start to caregiving may impact carers’ earning ability but could set the pattern for later-life economic stability or lack of it. On the other hand, older carers (65 years and older) also included a substantial number of respondents in our study, comprising 17.6% of CALD and 23.2% of non-CALD carers. Most older carers have considerable challenges, such as some element of ill health, financial vulnerability, and limited social support [37]. Our results corroborate the empirical evidence of the gendered nature of caregiving, with most carers being women [13,20,38,39,40]. The Australian governmental agencies report ‘near gender parity’ in workforce participation [41]. However, this perhaps does not include the more ‘hidden female population’ working fewer hours, as the pandemic and immediate post-pandemic period is one of economic vulnerability of considerable segments of the entire population. Also, social norms regarding familial roles are more focused on managing the imbalance in childcare responsibilities rather than a more comprehensive picture of the female role in caregiving for all familial responsibilities.

Our study also sought to identify key informal caregiving aspects for CALD and non-CALD respondents. The results showed that 80% of our respondents were primary carers in both groups and provided considerable assistance with mobility, self-care, and healthcare such as doctor visits, compliance with medical advice, etc. This is much higher than the 2018 triennial Survey of Disability, Ageing, and Carers (SDAC) in Australia, which found that about one in three or 33 percent of the respondents identified as primary carers [1]. The latest round of SDAC conducted in 2022 reported an increase in primary carers of 4 percent since 2018 [42]. It is difficult to interpret these results due to differences in survey methodology between SDAC 2018 and 2022 as well as the very specific questions asked in our study. There are no defined baseline estimates for the total number of family carers in Australia. The latest round of SDAC provides some indirect estimates. Note that SDAC uses a nationally representative sample, with results inferred in population-level estimates. Based on these estimates, there were approximately 3 million carers in 2022, of which 1.2 million were categorized as primary carers, whereas 383,600 were categorized as secondary carers. There is no clarity on the remaining 1.5 million carers regarding their role. With regard to CALD carers, there are no available statistics for the total number of family carers, as this information was not asked. It is therefore difficult to comment on the representativeness of the study sample.

Our results also found that non-CALD respondents engaged in more hours of weekly caregiving (37) than CALD carers (31) and were also providing care for more years (10) compared to CALD carers (7). These findings align with the broader research indicating that non-CALD caregivers often assume more intensive caregiving roles. This may be because non-CALD groups are likely to have lived in Australia for longer and may have more established family groups, e.g., parents and grandparents in Australia, who may be the care recipients. For instance, a 2020 report by Deloitte on informal care in Australia found that primary carers spend an average of 35 h of care per week, a sizeable portion exceeding 40 h. No further updated reports on this issue have been commissioned. The 2020 findings are consistent with our study’s findings that non-CALD caregivers tend to provide more hours of care weekly. Moreover, the duration of caregiving is a critical factor influencing caregiver burden and health outcomes. Research has shown that prolonged caregiving can increase physical and emotional strain. Our findings show that non-CALD caregivers provide care for more years (10 y) compared to CALD caregivers (7 y), suggesting that non-CALD caregivers may be at a higher risk for long-term health impacts. This is supported by studies indicating that extended caregiving periods are associated with higher rates of depression and a lower quality of life [8,43,44]. Additionally, the role of primary caregivers is crucial in understanding the dynamics of informal care. A report by Carers Australia (2020) [20] highlighted that primary caregivers often experience significant health challenges due to the demands of caregiving. Our study finds that 80 percent of respondents were primary carers, which underscores the need for targeted support and resources for these individuals, particularly in managing the intensity and duration of care.

National and international studies exploring informal carers’ health and well-being indicate an adverse impact of caregiving on physical and mental health [12,45]. We included several health measures: SF-12 general health and composite PCS and MCS scores, diagnosed chronic physical conditions, and depressive symptomatology. Nearly a third (29.9%) of carers reported their general health as fair/poor. The usual cut-off for satisfactory PCS is 50, and for MCS, values of less than 42 are interpreted as indicators of clinical depression [24,25]. For both CALD and non-CALD groups, the median PCS values were lower than 50, and the MCS score was lower than 42, indicating lower physical and mental health in our sample of informal carers. There is also a strong association between low MCS and depression. Depressive symptomatology has been associated with caregiving both by Australian [13,23,45] and international studies and reviews [12,46,47]. Though we had anticipated that depressive symptomatology would be more common in CALD respondents, we found that comparatively, the non-CALD carers experienced more depressive symptomatology. This group also had comparatively more chronic physical health conditions and lower median PCS and MCS scores. This could also be indirectly associated with respondents in the non-CALD group being older, having fewer secondary carers, more care hours, and longer duration of care.

The term CALD is unique to the Australian context. In relation to studies of immigrant populations, we briefly report on some studies from the US, Canada, and the UK that have large multicultural populations. A recent US study found that Asian family carers had relatively poorer physical and mental health compared to the non-carer Asian population. However, the health of Asian carers was similar to that of immigrant carers of various ethnicities, all of whom had poorer health compared to non-carers from their ethnic group [48]. Similarly, Canadian studies show the negative impact of relocation and caregiving roles for immigrant family carers [49]. In Britain and across Europe, the issues are also similar with regard to the adverse impact of caregiving on the health of family carers—both immigrant and non-immigrant participants. For example, data from large-scale longitudinal studies such as the English Longitudinal Study on Ageing and several waves of the European Survey of Health, Ageing, and Retirement show similar patterns [50], as observed in this present study for CALD and non-CALD carers. Similar findings regarding an adverse impact on the health of family carers have been reported from Singapore, which, although it has a smaller population than Western countries, has distinct native and immigrant groups [12].

With regard to this present study, it is difficult to infer causality through a cross-sectional study design. In longitudinal studies, there is a moderate impact on physical health due to long-term caregiving compared to non-caregivers [51]. The literature is clearer in relation to either developing or aggravating mental ill-health in relation to non-carers [51]. A large-scale longitudinal study on transition in caregiving roles by Lacey et al. (2024) [52] explored the caregiving health impact across various roles, from being a non-carer to becoming a carer (0–1 year), and longer-term caregiving (up to 8 years). They found that as caregiving responsibilities increased, the mental health of carers was adversely affected.

### Limitations

The cross-sectional design makes it difficult to draw causal inferences, particularly about physical and mental health issues, except that the SF-12 and CESD-12 scales assess physical and mental health in the four weeks preceding the survey. Although the diagnosis of chronic health conditions was prior to the survey, the lack of information on the time of diagnosis (pre- or post-carer role) makes it difficult to draw an association with caregiving burden. Within the CALD and non-CALD populations, there are sub-groups of family carers with a higher carer burden and added socioeconomic vulnerability, which makes them a ‘hard to reach’ population for survey research. Therefore, the adverse health impact could be higher, and their support needs could be more resource-intensive than is evident in this study. Other limitations include not having systematic information on the type of health issues of care recipients for which care was provided, which may impact carers’ health. We had initially included this question, but pilot testing showed that it was considered intrusive for an anonymous survey as, within certain ethnic communities, care recipients could be potentially identifiable.

## 5. Conclusions

In summary, our study found considerable similarities in both carer sub-groups in relation to the gendered nature of care, with women being primary carers, and substantial time being devoted weekly to care-related activities. Although sex and care hours were not statistically significant covariates for multivariable regression for PCS, there was an inverse association for the number of chronic physical health conditions and the MCS and CESD scores for both CALD and non-CALD carers. In terms of mental health as measured by MCS and CESD-12, a similar pattern was observed. In particular, being a female caregiver had a small but statistically significant association with CESD in non-CALD carers. The number of chronic physical health conditions seemed to strongly influence higher CESD scores in CALD carers, whereas MCS was important in both groups. With regards to the future, the increase in mental health-related disorders, increasing incidence of chronic health conditions, and ageing, it is important that caregivers receive early and adequate support for various issues associated with their own and care recipients’ health. With the rapidly increasing CALD population and ageing of some of the older immigrant communities, it is also imperative that support needs, both for carers and care recipients, are culturally appropriate rather than the usual policy of ‘one size fits all’. Furthermore, the paucity of research and limitations of cross-sectional surveys highlight the need to establish and adequately fund longitudinal studies to guide longer-term policy and program reforms.

## Figures and Tables

**Table 1:** Socio–demographic profile of CALD and non-CALD carers.

	CALD (n = 347)	Non-CALD (n = 302)	Statistical Significance
Demographic Characteristics	N (%)	N (%)	*p*-Value
Age			0.018
18–24	16 (4.6)	15 (5.0)	
25–34	74 (21.3)	52 (17.2)	
35–44	89 (25.7)	52 (17.2)	
45–54	65 (18.7))	57 (18.9)	
55–64	42 (12.1)	56 (18.5)	
65 and older	61 (17.6)	70 (23.2)	
Sex			0.189
Male	113 (32.6)	84 (27.8)	
Female	234 (67.4)	218 (72.2)	
Relationship status			<0.001
Single	72 (20.7)	80 (26.5)	
Married/Partner	242 (69.7)	169 (56.0)	
Divorced/Separated	10 (2.9)	10 (3.3)	
Widowed	23 (6.6)	43 (14.2)	
Education			<0.001
Primary	25 (7.2)	4 (1.3)	
Secondary	57 (16.4)	90 (29.8)	
Diploma/Technical	77 (22.2)	120 (39.7)	
University	187 (53.9)	88 (29.1)	
Adequacy of Financial Resources			0.148
More than adequate	44 (12.7)	58 (19.2)	
Adequate	90 (25.9)	84 (27.8)	
Occasionally adequate	163 (47.0)	124 (41.1)	
Often inadequate	42 (12.1)	29 (9.6)	
Do not know	8 (2.3)	7 (2.3)	
Born in Australia			<0.001
No	205 (59.1)	38 (12.6)	
Yes	140 (40.3)	264 (87.4)	
No response	2 (0.6)	----	
Years lived in Australia	N = 134 Mean: 14 ± SD, 17 Median: 21	N = 40 Mean: 40 ± SD, 16 Median: 47	<0.001

**Table 2:** Informal caregiving by CALD and non-CALD carers.

	CALD	Non-CALD	
Carer Characteristics	N (%)	N (%)	*p*-Value
Family size			<0.001
Up to two	65 (18.7)	23 (7.6)	
Between three and four	219 (63.1)	277 (91.7)	
Five or more	63 (18.2)	2 (0.7)	
Caregiver status			0.851
Primary	276 (79.5)	242 (80.1)	
Secondary	71 (20.5)	60 (19.9)	
Number of people needing long-term care	Mean 1.2, SD = 0.8,	Mean 0.89, SD = 0.78,	<0.001
Number of hours *p*/w devoted to caregiving activities (mean, SD, and median) *	30.8 ± 29.5 Median: 20	37.6 ± 36.7 Median: 25	0.011
Caregiving duration in years (mean, SD, and median)	7.3 ± 6.8 Median: 5.0	9.5 ± 9.0 Median: 6.0	<0.001
Number of other family carers			<0.001
Zero	140 (40. 3)	170 (56.3)	
One	138 (39.8)	96 (31.8)	
Two	46 (13.3)	23 (7.6)	
Three or more	22 (6.4)	13 (4.3)	
Location of other carers			<0.001
Same house	169 (48.7)	149 (49.3)	
Elsewhere	127 (36.6)	103 (34.1)	
Need interpreter services for care-recipient needs			<0.001
No	257 (74.1)	287 (95.0)	
Yes	90 (25.9)	15 (5.0)	

Note: * excludes respondents who reported a 24/7 carer role.

**Table 3:** Health status of CALD and non-CALD family carers.

	CALD	Non-CALD	
	N (%)	N (%)	*p*-Value
Self-rated health status			0.004
Excellent	37 (10.7)	18 (6.0)	
Very Good	81 (23.3)	70 (23.3)	
Good	141 (40.6)	108 (35.8)	
Fair	78 (22.5)	81 (26.8)	
Poor	10 (2.9)	25 (8.3)	
Number of diagnosed chronic health conditions *	N = 289	N = 275	0.078
Zero	107 (37.0)	74 (26.9)	
One	45 (15.6)	51 (18.5)	
Two	49 (17.0)	50 (18.2)	
Three or more	88 (30.4)	100 (36.4)	
Mean number (and SD) of chronic physical health conditions	2.19, SD = 2.94	2.09, SD = 1.96	0.652
Consulted doctor in last 6 months			0.029
No	92 (26.5)	65 (21.5)	
Yes	254 (73.2)	237 (78.5)	
Leave from paid work for health issues			<0.001
No	184 (53.0)	136 (45.0)	
Yes	132 (38.0)	60 (19.9)	
Not applicable	31 (8.9)	105 (34.8)	
Mean PCS [SF-12] score	45.5, SD = 10.6	44.8, SD = 11.0	0.431
Mean MCS [SF-12] score	35.1, SD = 11.3	35.6. SD = 14.0	0.607
Total CESD score (continuous)	8.8, SD = 5.1	9.2, SD = 5.8	0.313
Psychological health [CESD-12]			0.022
No depressive symptomatology (0–15)	255 (88.8)	249 (82.5)	
Moderate depressive symptomatology (16 or >)	92 (11.2)	53 (17.5)	

Notes: * totals do not equal 100% due to missing responses.

**Table 4:** Regression analysis for predictors of PCS, MCS, and CESD-12.

	CALD			Non-CALD	
**PCS**					
Model fit	AdjR^2^ = 0.30, *p* < 0.001			AdjR^2^ = 0.22, *p* < 0.001	
Variables	*std*β	*p*		*std*β	*p*
Age	−0.16	0.005		--	
Marital status	−0.12	0.032		--	
Care hours *p*/week	−0.14	0.009		--	
Health Conditions	−0.39	<0.001		−0.29	<0.001
MCS score	−0.22	<0.001		−0.29	<0.001
CESD	−0.25	<0.001		−0.46	<0.001
**MCS**					
Model fit	AdjR^2^ = 0.45, *p* < 0.001			AdjR^2^ = 0.60, *p* < 0.001	
Variables	*std*β	*p*		*std*β	*p*
Age	0.11	0.027		0.15	<0.001
No. of care recipients	0.12	0.010		--	
PCS score	−0.18	<0.001		−0.14	<0.001
CESD	−0.67	<0.001		−0.75	<0.001
**CESD**					
Model fit	AdjR^2^ = 0.50, *p* < 0.001			AdjR^2^ = 0.63, *p* < 0.001	
Variables	*std*β	*p*		*std*β	*p*
Sex	--	--		0.09	0.023
No. of care recipients	0.13	0.004		--	--
Primary carer	0.09	0.041		--	--
Health Conditions	0.14	0.004		0.89	0.022
PCS score	−0.18	<0.001		−0.23	<0.001
MCS score	−0.62	<0.001		−0.70	<0.001

## Data Availability

Restrictions apply to the datasets. The datasets presented in this article are not readily available, and requests to access the datasets should be directed to rafat.hussain@anu.edu.au.
